# Independent external validation of cardiovascular disease mortality in women utilising Framingham and SCORE risk models: a mortality follow-up study

**DOI:** 10.1186/1472-6874-14-118

**Published:** 2014-09-26

**Authors:** Louise Gek Huang Goh, Timothy Alexander Welborn, Satvinder Singh Dhaliwal

**Affiliations:** School of Public Health, Curtin Health Innovation Research Institute (CHIRI), Curtin University, Perth, Australia; Department of Endocrinology and Diabetes, Sir Charles Gairdner Hospital, Perth, Australia

**Keywords:** Cardiovascular disease, Risk estimation, Discrimination, Calibration, Model performance, Primary prevention

## Abstract

**Background:**

We conducted an independent external validation of three cardiovascular risk score models (Framingham risk score model and SCORE risk charts developed for low-risk regions and high-risk regions in Europe) on a prospective cohort of 4487 Australian women with no previous history of heart disease, diabetes or stroke. External validation is an important step to evaluate the performance of risk score models using discrimination and calibration measures to ensure their applicability beyond the settings in which they were developed.

**Methods:**

Ten year mortality follow-up of 4487 Australian adult women from the National Heart Foundation third Risk Factor Prevalence Study with no baseline history of heart disease, diabetes or stroke. The 10-year risk of cardiovascular mortality was calculated using the Framingham and SCORE models and the predictive accuracy of the three risk score models were assessed using both discrimination and calibration.

**Results:**

The discriminative ability of the Framingham and SCORE models were good (area under the curve > 0.85). Although all models overestimated the number of cardiovascular deaths by greater than 15%, the Hosmer-Lemeshow test indicated that the Framingham and SCORE-Low models were calibrated and hence suitable for predicting the 10-year cardiovascular mortality risk in this Australian population. An assessment of the treatment thresholds for each of the three models in identifying participants recommended for treatment were found to be inadequate, with low sensitivity and high specificity resulting from the high recommended thresholds. Lower treatment thresholds of 8.7% for the Framingham model, 0.8% for the SCORE-Low model and 1.3% for the SCORE-High model were identified for each model using the Youden index, at greater than 78% sensitivity and 80% specificity.

**Conclusions:**

Framingham risk score model and SCORE risk chart for low-risk regions are recommended for use in the Australian women population for predicting the 10-year cardiovascular mortality risk. These models demonstrate good discrimination and calibration performance. Lower treatment thresholds are proposed for better identification of individuals for treatment.

## Background

Cardiovascular disease (CVD) is one of the leading causes of death worldwide and in Australia [1, 2]. There were 45622 CVD deaths recorded in 2011, more deaths occurred in Australian females (23755) than Australian males (21867) [2]. Effective primary prevention strategies targeted at the individuals “at risk” are needed to reduce the number of CVD deaths [3]. The recent American College of Cardiology and American Heart Association guidelines [4] stress that both primary and secondary prevention should be directed at those most likely to benefit.

Multivariable CVD risk score models enable the quantification of CVD risk. These models determine the probability of an individual experiencing a CVD event within a predefined time period by assessing the entire risk-factor profile [5]. It identifies “at risk” individuals for intervention and is a cost-effective approach to CVD prevention [6]. The 2010 American College of Cardiology Foundation and American Heart Association Guidelines [7] recommends all asymptomatic women to undergo a global CVD risk assessment. CVD risk factors for women include age, race/ethnicity, obesity, poor diet, excessive alcohol consumption, physical inactivity, smoking, high blood pressure, high cholesterol, diabetes, chronic kidney disease, genetics, coronary artery calcium and psychological factors, and also the interactions between some of these risk factors [8, 9]. It should be noted that not all of these risk variables have been incorporated into risk score models. Recommended treatment thresholds are used to identify individuals at increased CVD risk for treatment.

Commonly used risk score models and treatment thresholds include, the Framingham risk score model for 10-year CVD incidence or death [10] (20% treatment threshold) [11, 12], SCORE risk chart for 10-year CVD death (3%, 5%, 7% and 10% treatment thresholds) [13], Reynolds risk score model for 10-year CVD incidence and death [14], ASSIGN risk score model for 10-year CVD incidence and death (20% treatment threshold) [12], general CVD risk score model for 10-year CVD incidence and death [15] (10% and 20% thresholds) [16, 17], simplified general CVD risk score model for 10-year CVD incidence and death [15] (10% and 20% thresholds) [16, 17] and QRISK score model for 10-year CVD incidence and death [18]. These risk score models were developed in the USA (Framingham, Reynolds and general CVD) and Europe (SCORE, ASSIGN and QRISK). Age, sex, systolic blood pressure (SBP), total cholesterol (TC) level, high-density lipoprotein cholesterol (HDL-C) level and smoking status were included in all six models. Diabetes status was included in all the models except the SCORE model. The Reynolds risk score model was the only model initially developed from a female population and it contains biomarkers in its calculation of the 10-year CVD risk [14]. The QRISK score model includes more risk variables i.e. body mass index (BMI) (which is also found in a simpler version of the general CVD risk score model), family history (also found in the Reynolds and ASSIGN model), Townsend deprivation score (a measure of social deprivation is also found in the ASSIGN model), use of antihypertensive medication (also found in the general CVD model), self-assigned ethnicity, rheumatoid arthritis, chronic renal disease and atrial fibrillation, compared with other models [18].

The Framingham and SCORE models were selected and validated in this study as they have similar endpoints. Both models predict 10-year CVD death risk. In addition, the risk variables used to calculate the 10-year CVD risk for the Framingham and SCORE models were collected in the National Heart Foundation (NHF) Risk Factor Prevalence Study. Some risk score models did not have recommended treatment thresholds for identifying women at increased risk of CVD and their performance could not be assessed.

Performance of risk score models is typically overestimated in the original data which they were developed [19]. External validation is an important step to evaluate the performance of risk score models using discrimination and calibration measures to ensure their applicability beyond the settings in which they were developed. The Framingham, SCORE and general CVD risk score models have been utilised to predict risk in an Australian population [20, 21]. Risk score models have also been developed for use in Australia i.e. from the Busselton Health Study and Dubbo Study. These models, however, are limited in applicability. The former model predicts the risk of coronary heart disease (CHD) hospitalisation or death while the latter predicts the CVD risk of older Australians aged 60 years and older [22, 23]. The National Vascular Disease Prevention Alliance recommends the use of the Framingham risk score model for estimating the 5-year CVD risk of disease-free Australians and an individual with a risk score of more than 15% is identified as high risk of developing CVD and is targeted for treatment [24].

The objective of this study was to conduct an independent external validation of the Framingham risk score model, SCORE model for low-risk regions (which was developed from European countries with low CVD rates: Belgium, Italy and Spain) and SCORE model for high-risk regions (European countries with high CVD rates: Denmark, Finland and Norway), using a mortality follow-up cohort of 4487 Australian women from the NHF Risk Factor Prevalence Study.

## Methods

### Study participants

Participants were selected from the NHF third Risk Factor Prevalence Study, 1989 [25]. Residents on the federal electoral rolls of December 1988 in North and South Sydney, Melbourne, Brisbane, Adelaide, Perth, Hobart, Darwin and Canberra were recruited for the Risk Factor Prevalence Study by systemic probability sampling of sex and 5-year age groups. A representative sample of 4487 women aged 20–69 years with no previous history of heart disease, diabetes or stroke at baseline were included for analysis. Participants taking medications to reduce their CVD risk factors were excluded.

### Ethics statement

Ethical approval for the NHF data was obtained in advance from the Australian Institute of Health Interim Ethics Committee, after consultation with the Commonwealth Privacy Commissioner. Participation was entirely voluntary. Those who participated signed an informed consent form [25]. Participant information was anonymised prior to analysis. This study was approved by the Human Research Ethics Committee at Curtin University. The linkage and analysis of the NHF data with the National Death Index were approved by the current Ethics Committee of the Australian Institute of Health and Welfare (AIHW), and complies with the Declaration of Helsinki.

### Risk factor measurements

A self-administered questionnaire was completed and information on demographic and clinical characteristics and smoking status were collected. Physical measurements were also taken: weight (to the nearest tenth of a kilogram), height (to the nearest centimetre), waist circumference and hip circumference were measured twice to the nearest centimetre according to standardised methodologies [26, 27], and systolic and diastolic blood pressure were recorded on the right arm of seated participants five minutes apart using mercury sphygmomanometers. The average of the two readings of blood pressure was used in the analysis [25]. Fasting blood samples were also collected in EDTA tubes and despatched to the central laboratory at the Division of Clinical Chemistry, Institute of Medical and Veterinary Science, Adelaide each week for cholesterol levels to be assayed. Participants were classified as non-smokers, previous smokers or current smokers [25].

### Cardiovascular disease outcomes

Mortality was ascertained to 31 December 1999 using the National Death Index maintained by the AIHW. The demographic data of the participants who were free from CVD and diabetes at baseline were submitted to AIHW. These data were then matched with the National Death Index using a probabilistic record linking package (“Automatch”). This provided data on the 10 year mortality follow-up. Causes of death were coded according to the International Classification of Diseases (ICD) 9^th^ or 10^th^ revision. ICD-9 codes 3900–4589 or ICD-10 codes I00.0-I99.9 were used for CVD deaths [28, 29]. The calculation of 10-year CVD risk from baseline data enabled assessment and comparison of the three risk score models and CVD death events were used because these events were determined, unlike non-fatal CVD events which are usually self-reported [9].

### Risk score models

The Framingham risk score model predicts the 10-year risk of CVD death [10]. It was developed using participants aged 30–74 years who were free of CVD and cancer from the American Framingham Heart Study. Age, sex, SBP, diastolic blood pressure, TC level, HDL-C level, smoking status and diabetes status were used in the calculation of the 10-year CVD risk [10]. Although electrocardiogram-left ventricular hypertrophy (ECG-LVH) is included in the Framingham model for the calculation of the 10-year CVD risk, participants were assumed not to have LVH as ECG was not undertaken in the NHF third Risk Factor Prevalence Study. The most commonly used treatment threshold for the Framingham risk score model was 20% [9]. This denotes that an individual who has a risk score of more than 20% is considered to be “at risk” of experiencing a CVD event within the next 10 years and should be targeted for treatment. Treatment includes lifestyle intervention (quit smoking, diet and exercise) as well as drug therapy (for lipids, hypertension and diabetes). Later versions of the Framingham risk score models include the National Cholesterol Education Program’s Third Adult Treatment Panel (ATP-III) risk assessment tool for the prediction of 10-year CHD risk, general CVD risk score model to predict the 10-year risk of CVD incidence and death and the Framingham risk prediction algorithm to quantify the 30-year risk of CVD [15, 30–33]. A new sex- and race-specific risk equation for predicting the 10-year atherosclerotic CVD risk, for fatal and non-fatal CVD events, for African-American and White men and women aged 40–79 years has also been published in the 2013 American College of Cardiology Foundation and American Heart Association Guidelines on the Assessment of Cardiovascular Risk [4].

The SCORE risk chart was developed by pooling 12 cohort studies in Europe. Participants aged 19–80 years with no previous history of heart attack were included in model development [13]. Cohorts in Denmark, Finland and Norway were used to develop the SCORE risk chart for high-risk regions as they reported higher CVD rates, adjusted for risk factor levels, age [34], cohort sizes and data availability while cohorts in Belgium, Italy and Spain were used for low-risk regions [13]. Fewer risk variables were used to calculate the 10-year risk of CVD death with the SCORE risk chart for low-risk and high-risk regions compared to the Framingham model. A larger cohort was used to develop the SCORE risk chart than the Framingham model. Age, sex, mean SBP, mean TC level, mean HDL-C level and smoking status were included in the calculation of the 10-year risk of CVD death for the SCORE models. Commonly used treatment thresholds for the SCORE risk chart included 3% and 5% for low-risk regions, and 3%, 5%, 7% and 10% for high-risk regions [13].

### Statistical analysis

The data on the representative sample of 4487 Australian females were described using mean ± standard deviation or median (inter-quartile range) for continuous variables, while counts and percentages were used for categorical variables. Ten-year predicted CVD mortality risk was calculated for each participant using the Framingham and the two SCORE risk models and these were compared against observed actual CVD mortality. The predictive accuracy of the CVD risk score models were assessed using both discrimination and calibration.

Calibration was assessed by ranking the cohort into quintiles of risk and comparing the number of predicted and observed deaths within each quintile. The Hosmer-Lemeshow chi-squared goodness-of-fit test was used to measure the agreement between predicted and observed events across the quintiles of predicted risk for each of the models [35]. A chi-squared *p*-value that is greater than 0.05 indicates a well-calibrated model. Graphically, calibration is represented as a plot of observed and predicted risk by quintiles of risk, arranged from low risk to high risk as determined by the CVD risk score models.

Discrimination was assessed by plotting the receiver operating characteristic (ROC) curve and calculating the area under the ROC curve (AUC) or c statistic. The ROC curve is a plot of true positives (sensitivity) against false positives (1-specificity) that provides a summary of sensitivity and specificity across a range of cut points for a continuous predictor [36]. The c statistic refers to the probability that the predicted risk is higher for a case than for a non-case and a c statistic of 1 indicates perfect discrimination and the predicted risks are higher for all cases than non-cases even if the predicted risk score differs from the observed risk [36–38]. Sensitivity, specificity, likelihood ratio and Youden index [39] were calculated at the respective recommended treatment thresholds of the risk score models, and at the proposed cut-off. P-values of less than 0.05 were considered to be statistically significant. All statistical analyses were performed with IBM SPSS Statistics Version 21.

## Results

The demographic and clinical characteristics of the cohort of 4487 women without baseline heart disease, diabetes or stroke are presented in Table 1. The predicted 10-year CVD death risk calculated using the Framingham risk score model, and SCORE risk chart for low-risk and high-risk regions are also presented. There were 152 deaths from all causes and 28 deaths due to CVD observed during the 10 years of follow-up. These 152 deaths represented approximately 3.4% of the sample and 0.3% of the total deaths in the Australian female population in 1989 [40], at the start of the study, and 0.2% in 1999 [41], at the end of the 10-year follow-up.

**Figure 1:**
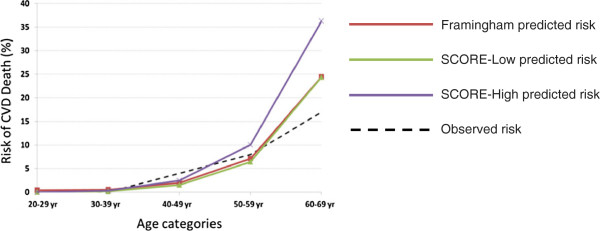
**Comparison of predicted and observed 10-year CVD death risk by age category.** (Framingham predicted risk – red line, SCORE-Low predicted risk – green line, SCORE-High predicted risk – purple line and observed risk – black dotted line).

In Figure 1, the observed 10-year CVD death risk is compared with the Framingham, SCORE-Low and SCORE-High predicted risks across the range of ages in the cohort. The risk score models generally underestimated the 10-year CVD death risk in those below 50 years of age and overestimated the risk in those aged 50 years and above. The Framingham risk score model and SCORE risk chart for low-risk regions predicted similar risk levels in the age categories while the SCORE-High model predicted higher risk levels compared to the other two models.

**Figure 2:**
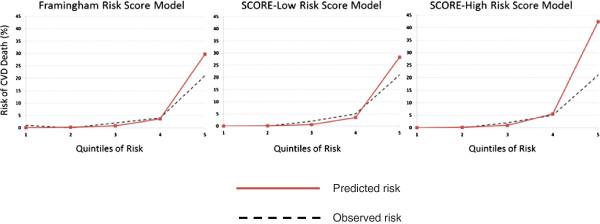
**Comparison of predicted and observed 10-year CVD death risk by quintiles of risk (Predicted risk – red line and observed risk – black dotted line).**

The Framingham, SCORE-Low and SCORE-High risk score models accurately predicted the 10-year risk of CVD death in the first four quintiles of risk (Figure 2). For the highest quintile of risk, however, all three models overestimated the 10-year risk. The overestimation was higher in the SCORE-High model than the Framingham and SCORE-Low models.

**Table 1:** **Characteristics of the cohort of 4487 women without heart disease, diabetes or stroke at baseline**

Variables	Total cohort	Survivors	Deaths (all-cause)	CVD deaths
	n = 4487	n = 4335	n = 152	n = 28
Age (years)	42.8 ± 13.2	42.3 ± 13.0	56.0 ± 11.9	60.8 ± 8.1
BMI (kg/m^2^)	24.8 ± 4.7	24.8 ± 4.7	26.2 ± 5.0	26.5 ± 3.8
WC (cm)	76.2 ± 11.1	76.0 ± 11.0	81.2 ± 10.7	82.5 ± 10.3
WHR	0.76 ± 0.06	0.76 ± 0.06	0.79 ± 0.07	0.80 ± 0.06
SBP (mmHg)	122.1 ± 18.4	121.6 ± 18.0	134.2 ± 23.4	143.7 ± 29.4
TC (mmol/L)	5.5 ± 1.2	5.4 ± 1.1	6.0 ± 1.2	6.5 ± 1.2
HDL-C (mmol/L)	1.5 ± 0.4	1.5 ± 0.4	1.5 ± 0.4	1.5 ± 0.4
Ratio of TC to HDL-C	3.9 ± 1.3	3.9 ± 1.3	4.3 ± 1.5	4.9 ± 2.2
Non-smoker	2652 (59.1%)	2570 (59.3%)	82 (54.0%)	13 (46.4%)
Previous smoker	880 (19.6%)	854 (19.7%)	26 (17.1%)	4 (14.3%)
Current smoker	955 (21.3%)	911 (21.0%)	44 (28.9%)	11 (39.3%)
FPR (%)	0.8 ± 1.8	0.7 ± 1.7	2.9 ± 3.7	5.2 ± 5.8
SCORE-Low (%)	0.7 ± 1.9	0.7 ± 1.6	3.1 ± 5.2	6.1 ± 9.9
SCORE-High (%)	1.1 ± 2.8	1.0 ± 2.4	4.5 ± 7.3	8.9 ± 13.8
FPR (%)	0.1 (0 , 0.6)	0.1 (0 , 0.5)	1.5 (0.2 , 4.1)	3.1 (1.0 , 6.6)
SCORE-Low (%)	0.1 (0 , 0.6)	0.1 (0 , 0.5)	1.8 (0.2 , 3.7)	2.9 (1.0 , 5.7)
SCORE-High (%)	0.1 (0 , 0.9)	0.1 (0 , 0.8)	2.8 (0.4 , 5.6)	4.3 (1.5 , 8.6)

Abbreviations: BMI, body mass index; WC, waist circumference; WHR, waist to hip ratio; SBP, systolic blood pressure; TC, total cholesterol; HDL-C, high-density lipoprotein cholesterol; FPR, Framingham 10-year predicted risk for CVD death; SCORE-Low, SCORE-Low 10-year predicted risk for CVD death; SCORE-High, SCORE-High 10-year predicted risk for CVD death.

Table 2 presents the discrimination and calibration model performance statistics for the Framingham, SCORE-Low and SCORE-High models. The ratio of the 10-year CVD predicted risk to the observed risk in the highest quintile was 1.41 for the Framingham model, 1.35 for the SCORE-Low model and 2.02 for the SCORE-High model. The observed number of deaths is 28 (Table 2). The Framingham risk score model predicted the total number of deaths to be 34.5, an overestimation of approximately 23%. The SCORE-Low model predicted the total number of deaths to be 32.5, an overestimation of about 16% while the SCORE-High model overestimated the number of CVD deaths by approximately 75%. Calibration, as assessed using the Hosmer-Lemeshow test, indicated that the overestimation was not significant for the Framingham and SCORE-Low models and that the models were calibrated. All models reported high ROC AUC (≥0.858).

**Table 2:** **Discrimination and calibration performance statistics for risk score models in estimating the 10-year risk of CVD death**

	FPR		SCORE-Low		SCORE-High	
Quintiles	Actual	Predicted	Actual	Predicted	Actual	Predicted
1	1^*^	0.1	0	0.0	0	0.0
2	0	0.3	0	0.1	0	0.2
3	2	0.8	2	0.6	2	1.0
4	4	3.6	5	3.5	5	5.6
5	21	29.7	21	28.3	21	42.4
Total	27	34.5	28	32.5	28	49.1
Hosmer-Lemeshow X^2^	4.74		6.09		12.06	
Hosmer-Lemeshow p-value	0.1918		0.1074		0.0072	
Calibrated model?	Yes		Yes		No	
ROC AUC (95% CI)	0.858 (0.786 – 0.929)		0.877 (0.827 – 0.927)		0.877 (0.827 – 0.927)	

*An outlier was excluded from quintile 1 (1 death) in the calculation of the Hosmer-Lemeshow test, owing to its undue influence on the test. Including this outlier would result in the Hosmer-Lemeshow X^2^ = 14.35, p-value = 0.0025, and the risk score model being assessed as not calibrated. This outlier did not represent a woman with elevated risk factors associated with increased CVD risk, on further examination.

Abbreviations: FPR, Framingham 10-year predicted risk for CVD death; SCORE-Low, SCORE-Low 10-year predicted risk for CVD death; SCORE-High, SCORE-High 10-year predicted risk for CVD death; ROC, receiver operating characteristic curve; AUC, area under the curve; CI, confidence interval.

Cut-points of the risk score models were identified from a review of the literature and their respective sensitivity, specificity, likelihood ratio and Youden index are reported (Table 3). Low sensitivities were observed for the cut-points from the literature, with the Framingham risk score model reporting 0% sensitivity at the 20% cut-point. Lower cut-points were identified at the maximal Youden index. The proposed cut-points are 8.7% for the Framingham model, 0.8% for the SCORE-Low model and 1.3% for the SCORE-High model, to identify those at an increased 10-year CVD death risk, at greater than 78% sensitivity and 80% specificity.

**Table 3:** **Diagnostic measures for the prediction of 10-year CVD death using Framingham, SCORE-Low and SCORE-High models**

Models	Cut-point determination	Cut-point	Sensitivity	Specificity	Likelihood ratio	Youden index
FPR	Literature [9]	20.0%	0.0%	100.0%	-	-
	Proposed*	8.7%	78.6%	81.1%	4.15	0.60
SCORE-Low	Literature [13]	3.0%	46.4%	93.7%	7.31	0.40
	Literature [13]	5.0%	28.6%	97.3%	10.72	0.26
	Proposed*	0.8%	82.1%	80.7%	4.27	0.63
SCORE-High	Literature [13]	3.0%	60.7%	89.5%	5.78	0.50
	Literature [13]	5.0%	35.7%	94.5%	6.47	0.30
	Literature [13]	7.0%	28.6%	96.9%	9.17	0.26
	Literature [13]	10.0%	17.9%	98.7%	14.01	0.17
	Proposed*	1.3%	82.1%	80.8%	4.29	0.63

Abbreviations: FPR, Framingham 10-year predicted risk for CVD death; SCORE-Low, SCORE-Low 10-year predicted risk for CVD death; SCORE-High, SCORE-High 10-year predicted risk for CVD death; proposed*, cut-point identified from our study using the Youden index for high sensitivity and specificity.

## Discussion

In this cohort of 4487 Australian females, the Framingham, SCORE-Low and SCORE-High models performed well in discriminating those who died from CVD from those who did not. However, only the Framingham and SCORE-Low models indicated that they were well-calibrated (*p* > 0.05) in this population, although risk was generally overestimated. The calibration statistics reported in previous study also indicated that the Framingham model was well-calibrated, consistent with our study, but not the SCORE risk chart [20].

Overestimation was observed in the highest risk quintile in our study, where the ratio of the 10-year CVD predicted risk to the observed risk was 1.41 for the Framingham risk score model, 1.35 for the SCORE-Low model and 2.02 for the SCORE-High model. It had also been suggested previously that the accuracy of risk prediction at the extremes of risk needs to be improved [42]. A systematic review study on the external validation of the Framingham risk score model for predicting the 10-year CHD or CVD risk reported that the ratio of 10-year CHD predicted to observed risk ranged from an under-prediction of 0.43 in a high-risk population to an over-prediction of 2.87 in a lower-risk population [43]. There was a similar trend for the prediction of 10-year CVD risk [43]. An under-prediction of risk was reported in Malaysian women using the SCORE risk chart [44].

In our study, the Framingham risk score model overestimated the total CVD deaths by 23%, the SCORE-Low model overestimated by about 16% and the SCORE-High model overestimated the number of CVD deaths by approximately 75%. In a study of Australian men free of heart disease, stroke and diabetes, the Framingham risk score model significantly over-predicted the risk of CHD and CVD death in all quintiles [45]. A study on two German prospective cohorts found that the Framingham predicted risk substantially exceeded the observed risk in the cohorts, with an overestimation of at least 50% [46]. An overestimation of risk was also observed in another study, the expected CHD deaths was 162, however, only 148 CHD deaths were observed [47]. A study which compared the performance of the Framingham model and the SCORE model in predicting the 10-year CHD and CVD mortality risk in a non-diabetic population aged 40–65 years from a Spanish healthcare centre found that the SCORE model over-predicted the risk by 40% while the Framingham model over-predicted the risk by 64% (33% in men and 150% in women) [48]. Using the SCORE risk chart for low-risk regions to predict the 10-year risk of CVD death, a study observed that the model over-estimated the CVD mortality risk in women [49]. Overestimation was also reported for the SCORE risk chart for high-risk regions and the overestimation increased with age in women from Norway, consistent with our study findings [50]. Similarly, the Framingham risk score model over-predicted the 5-year CVD risk (*p* < 0.05) in the Australian Diabetes, Obesity and Lifestyle study, evaluated against the general CVD risk score model [21]. The risk was also overestimated using the general CVD risk score model. Another study assessed the performance of the Framingham risk score model and a general practice model in a cohort of 3582 women aged 60–79 years who were initially free of CHD in the British Women’s Heart and Health Study and observed that the Framingham model accurately predicted the 5-year CHD risk but over-predicted the CVD risk [51]. Similar to our study, the model overestimated CVD risk in higher-risk groups. The Framingham risk score model also over-predicted the 10-year CVD risk in another study by 35% [52]. It over-estimated the risk less than the ASSIGN model but more than the QRISK model. Its performance was also compared with the QRISK score model in two United Kingdom (UK) cohorts and the Framingham model over-predicted the risk as well [53].

Similar to our study findings, a systematic review study which assessed the performance of the Framingham risk score model using 25 validation cohorts of different population groups in the analysis reported that it accurately predicted CHD risk in populations from the United States, Australia and New Zealand [54]. In the Dubbo study, the Framingham risk score model for CHD risk also accurately predicted the 10-year CHD incidence in men and women aged 60–79 years who were free of CVD or diabetes at baseline [22]. Another study comparing the performance of ASSIGN, Framingham and general CVD risk score models in predicting the 10-year CVD risk in a UK cohort of patients from general practice reported that the Framingham model predicted the risk accurately but some over-prediction was observed in women of higher risk [55]. The Framingham CVD model was also compared with the ATP-III score and Reynolds risk score using a case-cohort sample of the multi-ethnic Women’s Health Initiative Observational Cohort. Contrary to our study findings, the calibration performance of the Framingham CVD and ATP-III models were poor, however, the Reynolds model was calibrated [56].

It is important to extensively evaluate the applicability of risk models to each population, in order to avoid under-prediction or over-prediction [57]. Possible causes of differences in estimation include differences in the populations used to develop the risk score models and different risk variables used to estimate risk. Generally, age, sex, SBP, TC level, HDL-C level and smoking status are included into risk score models. The incorporation of additional risk variables such as race/ethnicity, obesity, poor diet, excessive alcohol consumption, physical inactivity, chronic kidney disease, family history of CVD, coronary artery calcium and psychological factors, and also interactions between some of these risk factors could improve risk estimation in women. Recalibration has also been shown to improve the performance of risk score models after accounting for differences in the prevalence of risk factors and underlying CVD rates [58, 59]. Another option would be to quantify the amount of overestimation and adjust accordingly [46].

Existing studies generally reported good discrimination, consistent with our study findings. Our study reported AUC ≥ 0.858. High AUC (0.866) has also been reported for the Framingham risk score model in predicting CVD death in Australian men and women [60]. AUC of 0.880 and 0.770 were also reported in women using the Framingham risk score model for predicting myocardial infarction and CHD death, in two German prospective studies [46]. A study also found that the SCORE risk chart for low-risk regions for predicting the 10-year risk of CVD death discriminated Austrian women well (AUC = 0.780) [49]. The discrimination performance of the SCORE risk chart for high-risk regions to predict the 10-year CVD mortality was also evaluated in Norway and the AUC values ranged from 0.660 to 0.720 in women [50]. Another study which assessed the validity and utility of cardiovascular risk score models in a Malaysian population found that the Framingham risk score model and SCORE risk chart for low-risk and high-risk regions demonstrated good discrimination for predicting the 5-year cardiovascular mortality risk (AUC ranged from 0.758 to 0.763) in women [44].

The discriminative ability of the Framingham risk score model and SCORE risk chart have also been compared to other models. The Framingham risk score model was evaluated against the general CVD risk score model in the Australian Diabetes, Obesity and Lifestyle study and reported an AUC of 0.740, which is lower than the general CVD risk score model (AUC *=* 0.760) [21]. Another study compared the performance of the Framingham, QRISK and ASSIGN models and reported that the Framingham model had lower ROC statistic (0.774) in women than QRISK and ASSIGN models [52]. Similar results were observed in two UK cohorts with the Framingham risk score model reporting lower ROC statistics (approximately 0.776 and 0.760) than the QRISK score model [53]. The performance of the Framingham CVD model was also compared with the ATP-III score and Reynolds risk score in a multi-ethnic Women’s Health Initiative Observational Cohort. The c statistics of the Framingham model and ATP-III score were 0.750 and 0.757, respectively, and were both lower than the Reynolds model [56].

The threshold discrimination is also an important aspect to consider, particularly in clinical practice [61]. The sensitivity and specificity of CVD risk score models at different cut points or treatment thresholds should be reported [62]. In our study, participants at increased risk of CVD were not adequately identified for treatment at the respective recommended treatment thresholds of the three risk score models. Low sensitivity and high specificity were reported as the recommended treatment thresholds were high. The higher treatment thresholds currently used for risk score models, under-treat individuals in higher risk groups. Our study identified lower treatment thresholds for the Framingham risk score model (8.7%) and SCORE risk chart for low- (0.8%) and high-risk (1.3%) regions, to improve the identification of individuals who require treatment with high sensitivity and specificity. Previous studies have also reported that it is difficult for individuals to exceed the recommended treatment thresholds even with markedly elevated risk factors. The Framingham risk score model was compared against the ASSIGN risk score using the Scottish Heart Health Extended Cohort. At the 20% cut point, the sensitivity of the Framingham risk score model was low (45.6%), while the specificity was 82.5% [12]. In another study, the Framingham model for predicting the 10-year CHD risk classified only 33% of all CHD events occurred in women as high risk, using a threshold of 20% [63]. The Framingham risk score model and SCORE model to predict CVD mortality was also evaluated in the Netherlands and the Framingham model only identified 0.7% of the population for treatment while the SCORE model assigned 0.4% for treatment, at a threshold of 10% [64]. Among those identified for treatment using the Framingham and SCORE models, their observed risk was 6.2% and 10.2%, respectively, indicating that the recommended threshold was inadequate for identifying individuals for treatment. The 10-year predicted risk was < 10% or low across age groups and risk factor levels using the ATP-III risk assessment tool [32]. Similarly, all men < 30 years with substantial risk factor burden using the Framingham risk score model also reported low risk using existing threshold [65]. In addition, the 10-year predicted risk was also found to be < 10% in most men < 50 years and most women < 70 years [66]. An approach to lower treatment thresholds would seem appropriate. A new threshold of ≥ 10% has been recommended for identifying women at high CVD risk for treatment as it is difficult for a woman below 75 years of age with several markedly elevated risk factors to exceed a 10% (let alone a 20%) with the ATP-III risk assessment tool [16]. The most recent guidelines released by the American College of Cardiology and American Heart Association Task Force on Practice Guidelines have recommended an even lower threshold of 7.5% for the 10-year risk of atherosclerotic CVD for identifying individuals for treatment [4]. Lower treatment thresholds, as proposed, would improve the identification of individuals who require treatment, at the expense of increasing the numbers to treat and increasing associated costs. Determining thresholds using diagnostic measures to maximise sensitivity and specificity is still the preferred approach.

### Study strengths and limitations

Our study has limitations. The endpoints of our study were fatal CVD events established from death certificates and no incidence data was available. There were only 28 fatal CVD events among 4487 women in the 10-year follow-up. It is possible that calibration defaults are present due to differences in the populations used to develop the Framingham and SCORE models. Though differences exist, these models have continued to exhibit calibration when externally validated against other populations and have been used extensively in the assessment of CVD risk in previous studies [20, 22, 54, 55]. Participants were assumed not to have LVH as ECG was not undertaken in the NHF third Risk Factor Prevalence Study and this could affect risk prediction of the Framingham risk score model. It has been reported that while baseline ECG data to determine LVH were not available, this is unlikely to have a significant impact on risk prediction as ECG-diagnosed LVH is very rare in the general population without CVD [67]. Only one set of baseline measurements was recorded for some risk variables but important variables were measured twice. Menopausal status of women was not recorded at time of survey. Low number of CVD events limits the ability for further detailed analyses to assess other risk factors currently not included in the risk score models discussed.

## Conclusions

In this study, we have provided an independent external validation of the Framingham risk score model and the SCORE risk chart for low-risk and high-risk regions on a cohort of 4487 Australian females. The use of the Framingham risk score model and SCORE risk chart for low-risk regions, for predicting the 10-year CVD mortality risk in the Australian women population is recommended. These models demonstrate good discrimination and calibration performance. Lower treatment thresholds are needed, in order to better identify individuals for treatment.

## Abbreviations

CVD: Cardiovascular disease
CHD: Coronary heart disease
NHF: National Heart Foundation
AIHW: Australian Institute of Health and Welfare
ICD: International Classification of Diseases
SBP: Systolic blood pressure
ECG-LVH: Electrocardiogram-left ventricular hypertrophy
ATP-III: National Cholesterol Education Program’s Third Adult Treatment Panel
ROC: Receiver operating characteristic curve
AUC: Area under the curve
UK: United Kingdom
BMI: Body mass index
WC: Waist circumference
WHR: Waist to hip ratio
TC: Total cholesterol
HDL-C: High-density lipoprotein cholesterol
FPR: Framingham 10-year predicted risk for CVD death
SCORE-Low: SCORE-Low 10-year predicted risk for CVD death
SCORE-High: SCORE-High 10-year predicted risk for CVD death
CI: Confidence interval
Proposed^*^: Cut-point identified from our study using the Youden index for high sensitivity and specificity.
